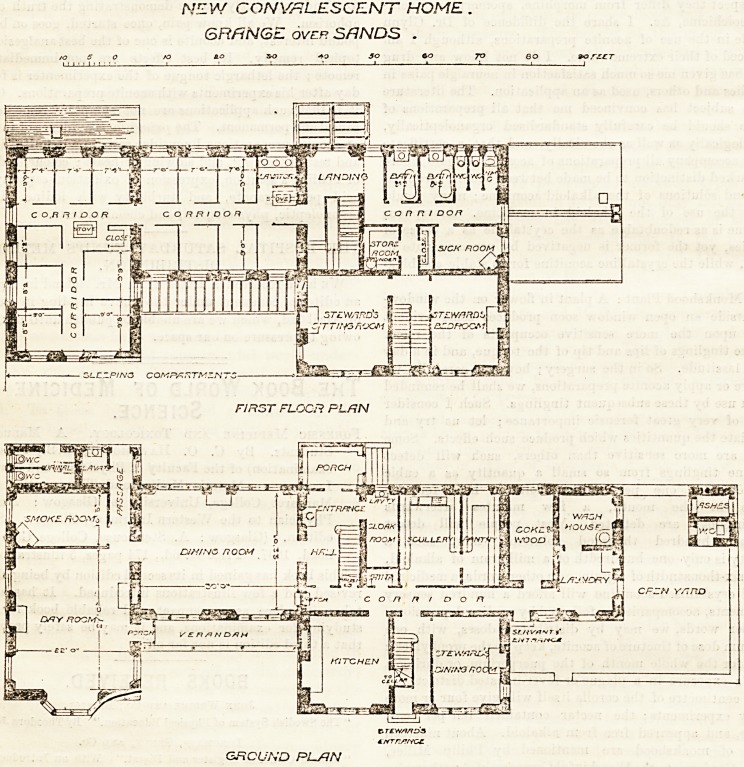# Hospital Construction

**Published:** 1897-04-10

**Authors:** 


### The Institutional Workshop.

### HOSPITAL CONSTRUCTION.

### THE CONVALESCENT HOME, GRANGE-OVER-SANDS, LANCASHIRE.

This home, in course of erection, stands in pleasant grounds of two acres, overlooking the sea, its principal front facing south-east. The building is intended to have two wings, each of three storeys above ground, with an administration block between them, but only one wing is at present in hand. The central block contains, on the ground floor, an entrance-hall and corridor, which, as shown on the plan, give access to the male patients' dining-room, from which the day-room and smoking-room are approached. The kitchen and steward's room are in front of the corridor, and the scullery and pantry behind. The steward has a separate staircase leading to a second sitting-room and three bedrooms on the upper floors, while the main staircase rises from the entrance-hall, and give access on the first floor to two dormitories in the wing, and to the bath-rooms, closets, store-rooms, and "sick-room" in the central block. The dormitories contain 15 cubicles, with partitions 7 feet high, and a window to each, and the whole arrangement is repeated on the second floor, making 30 beds in all. Two points are noticeable in the plans: the "sick-rooms" are very close to the closets, and the ground floor corridor seems to have very little light or ventilation. The use of the dining-room as a passage to the day-rooms is also undesirable, and it might easily have been avoided.

The principal rooms are warmed by ventilating stoves; and vertical inlet tubes, with exhaust flues leading to ventilators on the roofs, are also provided for changing the air.

The building is being built for the friendly societies of the North-Eastern counties, and the cost is stated as about £3,000, less than £100 per bed, when the administration block is taken into consideration. The architect is Mr. John Hutton, of Kendal, from whose drawings the plans are reproduced.

## Figures and Tables

**Figure f1:**